# In Vitro Degradation and Cytotoxicity of Eggshell-Based Hydroxyapatite: A Systematic Review and Meta-Analysis

**DOI:** 10.3390/polym13193223

**Published:** 2021-09-23

**Authors:** Rohmadi Rohmadi, Widyanita Harwijayanti, Ubaidillah Ubaidillah, Joko Triyono, Kuncoro Diharjo, Pamudji Utomo

**Affiliations:** 1 Mechanical Engineering Department, Faculty of Engineering, Universitas Sebelas Maret, Jalan Ir. Sutami 36A, Kentingan, Surakarta 57126, Indonesia; rohmadi@student.uns.ac.id (R.R.); widyanitaharw@student.uns.ac.id (W.H.); jokotri5528@gmail.com (J.T.); kuncorodiharjo@ft.uns.ac.id (K.D.); 2 Department Orthopaedic Traumatology, Prof Dr. R. Soeharso Orthopaedic Hospital Surakarta/Faculty of Medicine, Universitas Sebelas Maret Jalan Ir. Sutami 36A, Kentingan, Surakarta 57126, Indonesia; pamudji_utomo@staff.uns.ac.id

**Keywords:** eggshells, hydroxyapatite, degradation, cytotoxicity, meta-analysis

## Abstract

Objective: This review focuses on the in vitro degradation of eggshell-based hydroxyapatite for analyzing the weight loss of hydroxyapatite when applied in the human body. Cytotoxicity tests were used to observe cell growth and morphological effects. A systematic review and meta-analysis were conducted to observe the weight loss and viable cells of hydroxyapatite when used for implants. Method: Based on the Population, Intervention, Comparison, and Outcome (PICO) strategy, the articles used for literature review were published in English on SCOPUS, PubMed, and Google Scholar from 1 January 2012 to 22 May 2021. Data regarding existing experiments in the literature articles the in vitro degradation and cytotoxicity testing of eggshell-based hydroxyapatite determined the biocompatibility of the materials. A meta-analysis was conducted to calculate the mean difference between the solutions and soaking times used for degradation and the stem cells used for cytotoxicity. Results: From 231 relevant studies, 71 were chosen for full-text analysis, out of which 33 articles met the inclusion criteria for degradation and cytotoxicity analysis. A manual search of the field of study resulted in three additional articles. Thus, 36 articles were included in this systematic review. Significance: The aim of this study was to highlight the importance of the biocompatibility of eggshell-based hydroxyapatite. The weight loss and viability cells of eggshell-based hydroxyapatite showed optimum results for viable cells requirements above 70%, and there is a weight loss of eggshell-based hydroxyapatite for a material implant. The meta-analysis indicated significant differences in the weight loss of eggshell-based hydroxyapatite materials with different soaking times and solutions used. The various kinds of stem cells for incubation of cultured cells in contact with a device, either directly or through diffusions with various kinds of stem cells from animals and humans, yielded viability cells above 70%.

## 1. Introduction

Bone defects due to fractures remain a challenge for clinicians to repair. Recovery usually requires the use of synthetic biomaterials [1]. Accidents and chronic illnesses cause bone defects that do not heal on their own, so the development of more efficient treatments is needed [2]. Porous biomaterials are scaffolds that are suitable for forming bone tissue and encouraging the diffusion of nutrients and metabolites out of cells and scaffolds [3]. Scaffolds can be absorbed throughout the body of living things, and diffusion of the scaffold substance involves all bone tissues, whether they are in good health or are seriously damaged [4]. Hydroxyapatite, as a bioactive ceramic material, is the best candidate for bone tissue replacement among the variety of biomaterials [5]. Some researchers believe that the use of calcium from natural sources can reduce the risk of health issues and leads to better cell proliferation [6].

Millions of tons of shell waste (seashells, eggshells, etc.) are discarded after their contents are consumed as food [7,8]. Shell waste is a substantial source of calcium because most of the content contained in this material is calcite (CaCO_3_) [9]. However, some marine species, such as oysters, are in danger of extinction, and most of these species have a slow growth rate. Thus, the selection of new alternative materials that are renewable, inexpensive, and easy to locate is highly recommended [10]. An important process is recycling waste into useful products. Eggshells are one of the sources of organic shell waste that are disposed of daily [11]. Development of the method for converting eggshell into hydroxyapatite is very important. Eggshells form approximately 11% of the total egg weight, consisting of 94% calcium carbonate (CaCO_3_), 1% calcium phosphate (Ca_3_(PO_4_)_2_), and 4% organic matter [11,12,13,14,15]. Eggshells also contain many trace elements, such as Na, Mg, and Sr, resembling the bone matrix in humans [16].

Hydroxyapatite is a hydrated calcium phosphate from the mineral group of apatite. Its chemical formula is Ca_10_(PO_4_)_6_(OH)_2_ and it has a Ca/P ratio of 1.67 [8,17,18]. HA is a compound that has a hexagonal structure with white solids [19]. Because it has biocompatibility, bioactivity [20,21,22] and significant osteoinductivity [23], hydroxyapatite has been widely used as a bone substitute to fill bone defects, such as matrix scaffolding for tissue engineering [24] and as a coating on biomedical implants [20]. In biomedical applications, biomaterials must have reliable biocompatibility capabilities [25]. Biomaterials should not release any toxins or produce harmful reactions in the human body. Cytotoxicity is related to the degradation process of biomaterials, which can stimulate or inhibit the metabolism of cultured stem cells [26]; biomaterial degradation is affected by porous size and porous interconnectivity [27].

Ganesan et al. soaked hydroxyapatite in phosphate buffer saline (PBS) to evaluate in vitro degradation by measuring the weight loss of eggshell-based hydroxyapatite [27]. Kumar et al. performed a cytotoxicity test on hydroxyapatite using mouse fibroblast 3T3-L1 cells with variable observation times [20]. Horta et al. investigated eggshell-based hydroxyapatite cells with 98.9% viability that were cultured in dental pulp stem cells (DPSCs) for 24 h [26]. Both in vitro degradation and cytotoxicity tests were carried out to determine the biocompatibility of the materials. However, meta-analyses of the in vitro degradation and cytotoxicity from eggshell-based hydroxyapatite are limited, and also the effects of soaking time variations, solutions used, cultured stem cells, and the influence of elements added to the composites in the existing published literature may not yet be displayed.

The aim of this study was to present a systematic review and meta-analysis of studies that have investigated the in vitro degradation and cytotoxicity of eggshell-based hydroxyapatite. This meta-analysis was designed to determine the effects of soaking time and solutions used for the degradation of eggshell-based hydroxyapatite and to explore the influence of different viability stem cells and the morphology of the eggshell-based hydroxyapatite that are described in published results.

## 2. Materials and Methods

### 2.1. Study Design

This systematic review and meta-analysis were performed according to the PRISMA (Preferred Reporting Items for Systematic Reviews and Meta-Analysis) guidelines. The PICO strategy involved the following:

- Population—biological evaluation, including in vitro degradation and cytotoxicity test;
- Intervention— medical implants;
- Comparison—soaking times and solutions used for degradation testing, together with different stem cells cultured for cytotoxicity testing;
- Outcomes—effects of the in vitro degradation and cytotoxicity test results to highlight the biocompatibility of eggshell-based hydroxyapatite.

### 2.2. Information Sources

The search for articles was carried out in the SCOPUS, PubMed, and Google Scholar databases. All the articles were published in English between 1 January 2012 and 22 May 2021. After screening based on the inclusion criteria for degradation and cytotoxicity analysis for each article, they were all imported to Mendeley Desktop. The articles are focused on eggshells as a source of hydroxyapatite, and also mixing the shells with other materials, such as polycaprolactone, rice husk ash, selenium (Se), chitosan, silicon, hair keratin, and jellyfish collagen. To complement the electronic search, a manual search was conducted in the following principal periodicals specific to the field: *International Journal of Applied Ceramic Technology*, *Journal of Materials Science*, *Materials in Medicine*, *International Journal of Nanomedicine*, *Journal of the American Ceramic Society*, *Ceramics International*, *Materials Science and Engineering C*, *Biomedical Materials*, *Composites Part B, Journal of Sol-Gel Science and Technology*, *Colloids and Surfaces B: Biointerfaces, Nanomaterials*, *Materials Research*, *Chemistry Select*, *Journal of Biomedical Materials Research: Part A*, *Journal of International Oral Health*, *ACS Biomaterials Science and Engineering*, *Bulletin of Material Science*, *Polymers*, *Journal of Biosystems Engineering*, *Society for Biomaterials*, *International Journal of Dentistry*, *Royal Society for Chemistry*, *International Journal of Biological Macromolecules*, and the *Journal of Materials Research and Technology*. The last search was performed on 22 May 2021.

The search terms and their combinations were “hydroxyapatite”, “eggshells” and “hydroxyapatite”, “eggshells” and ”hydroxyapatite” and ”biocompatibility”, “eggshells” and ”hydroxyapatite” and ”degradation”, “eggshells” and ”hydroxyapatite”, and ”cytotoxicity”. The title, abstract, and keywords were included in the search terms. From the three databases used, a total of 33 articles and three additional articles from manual search within the specific field were included in this systematic review.

### 2.3. Inclusion and Exclusion Criteria

Biocompatibility studies reporting on the in vitro degradation and cytotoxicity testing of eggshell-based hydroxyapatite were included. Publications were excluded if the studies included other biocompatibility testing such as bioactivity, drug delivery, antibacterial, or hemolysis testing.

### 2.4. Data Extraction and Collection

The data from the articles of interest were tabulated by compiling a spreadsheet in Excel software (Microsoft Corp., Redmond, Washington, United States). The following data were collected from the included articles: demographic information (author, journal, title, date), sample preparation and composition (eggshell-based hydroxyapatite), testing preparation (soaking times and solutions used for in vitro degradation, together with stem cells for cytotoxicity), and the results of in vitro degradation and cytotoxicity testing (weight loss, viable cells, and morphology of the materials).

### 2.5. Data Analysis

The material composition and method variability of the studies included were used for the meta-analysis. For the meta-analysis, a statistical analysis was conducted based on results from articles, including the various samples which were analyzed for soaking times, solutions used, and different stem cells used for culturing. The weight losses of the materials were plotted on graphs. The results for various stem cells are presented in Table 2. The template of viable cells for each testing method was copied from each article and is presented in Tables 3 and 4. In addition, analyses were performed to assess the different results from the cytotoxicity testing methods used for eggshell-based hydroxyapatite. The data are displayed in the form of qualitative and quantitative results.

## 3. Results

### 3.1. Study and Information Selection

**Figure 1:**
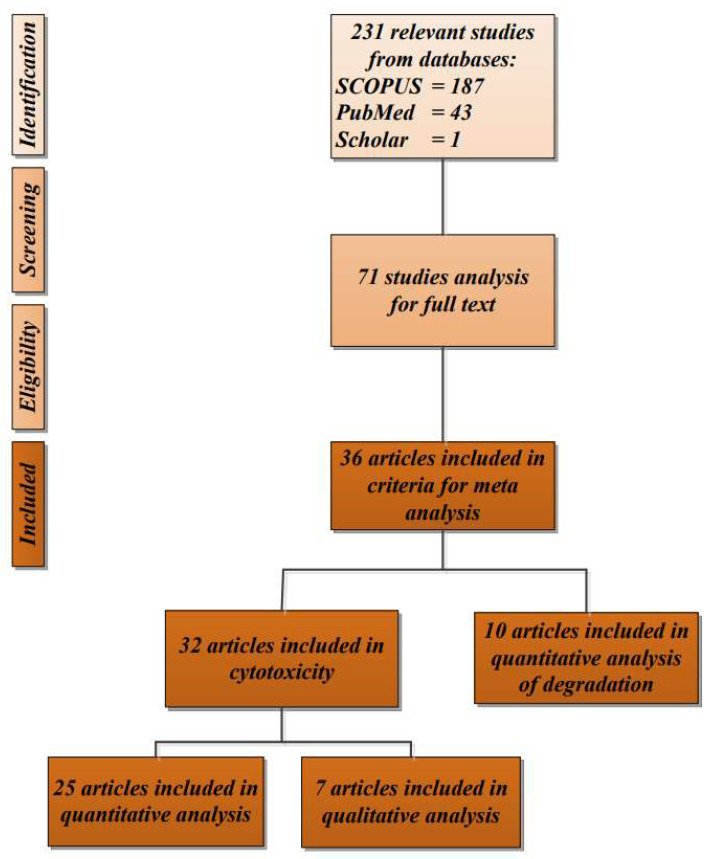
Flow diagram of the study selection of the articles included in the systematic review.

Out of 71 relevant article titles collected from SCOPUS, PubMed, and Google Scholar, only 36 were included after screening the title, abstract, and content for in vitro degradation and cytotoxicity testing for the biocompatibility of eggshell-based hydroxyapatite. The articles came from several countries, such as India [11,14,27,28,29,30,31,32,33,34,35,36,37,38,39,40], Bangladesh [41,42], China [43], Korea [25,44,45], Turkey [46,47,48], Thailand [49,50], Brazil [26], Italy [51], USA [52], Romania [2], Vietnam [53,54], Colombia [55], and Egypt [56]. The study selection of the articles included in the systematic review is shown in Figure 1. The articles were published from 1 January 2012 to 22 May 2021.

**Figure 2:**
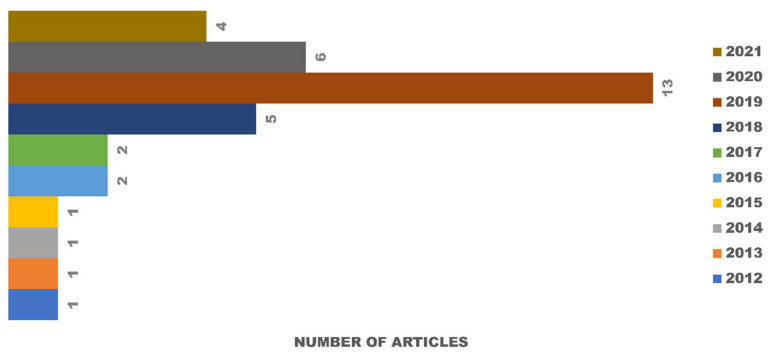
Articles published on the subject of in vitro degradation and cytotoxicity testing of eggshell-based hydroxyapatite from 1 January 2012 to 22 May 2021.

Out of 71 articles collected for full-text analysis from SCOPUS, PubMed, and Google Scholar, only 36 studies were included after applying the inclusion and exclusion criteria. Three studies were excluded because the results of the cytotoxicity tests were not specific and used different methods of measurement. During 2012–2016, only 6 articles were published, resulting in a mean of 1.2 articles per year. In the next five years (2017–2021), the field rapidly grew, and a total of 30 articles were published in this period (Figure 2).

### 3.2. Qualitative Analysis

**Figure 3:**
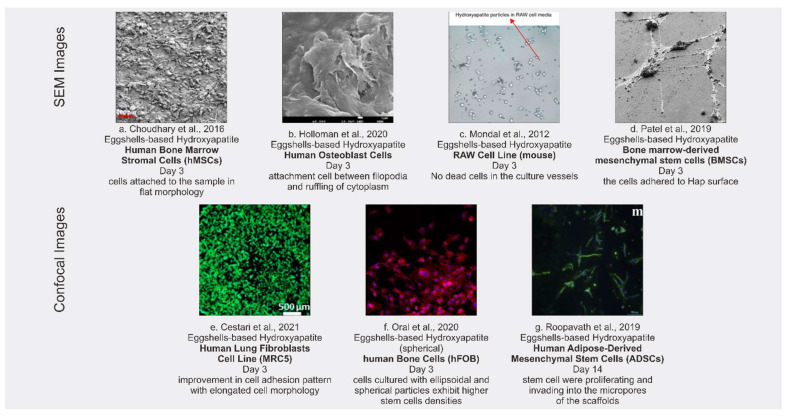
SEM images (**a**–**d**) and confocal images (**e**–**g**) of cytotoxicity tests with different stem cells for cultures.

All the articles included pertain to in vitro degradation and cytotoxicity tests of eggshell-based hydroxyapatite. The results of the cytotoxicity testing consisted of two types: quantitative and qualitative. The results of the quantitative testing are discussed in Section 3.3. The qualitative results are displayed in the Figure 3, showing the appearance of the surface morphology of the materials. Of the articles included in this review, the qualitative results involved seven cytotoxicity tests, from the articles of [32,34,38,44,48,51,52].

### 3.3. Meta-Analysis

Eggs are a major source of food consumed by people worldwide [12]. There are different eggshell types used to obtain hydroxyapatite, such as chicken eggshell [57,58,59], avian eggshell [13], and duck eggshell [60]. Chicken eggshells are the most abundant raw material used to produce hydroxyapatite [57]. The shape and crystalline structure of hydroxyapatite play important roles in mediating cellular behaviors and/or tissue functions in human bones [25]. There are different techniques for obtaining hydroxyapatite, such as the wet precipitation method [61,62], sol-gel method [4], hydrothermal method [18], solid-state method [10,14], microwave irradiation [11], and the sonochemical method [37]. In the case of bone regeneration, scaffolds must possess other important properties, such as osseointegration and osteoconduction. Their main role is to facilitate the formation of an extracellular matrix [2]. In vitro biological tissue formation is have potential to be used as replacement for animals along with pharmacological and toxicological screening, and as a replacement tissue for use in clinical applications [63].

For in vitro degradation and cytotoxicity tests, a meta-analysis was performed using experimental data from 36 literature articles, and the results are described in Table 1 and Table 2. Table 1 shows the results obtained from studies reporting on solutions and soaking times used to observe the weight loss of the eggshell-based hydroxyapatite compositions. Weight loss analysis is affected by the number of days and solutions used during the soaking process, along with the composition of the material. Weight loss can also be observed by the purity and shape of the hydroxyapatite powder, microstructure of hydroxyapatite [31], the condition of the human body, or the solutions used to adjust to the pH of the human body [27]. The observed porosity of the microstructure hydroxyapatite affects the absorption of the solution used due to the surface area of eggshell-based hydroxyapatite [27]. The formation of a secondary phase from hydroxyapatite to β-TCP also affects the releasing ion [27]. The percentage of weight loss is given by the result obtained from degradation testing. A degradation test is carried out by soaking the materials using a solution with the same pH as the human body. Samples are immersed in solutions and incubated at 37 °C—the typical human body temperature—for a period of time, between 7 until 28 days [27]. The solutions are refreshed at certain times, after 1 h for the first 5 h, and then every 24 h after first time soaking, and on the 7th day the samples are removed and dried [35]. The samples are weighed and the percentage of weight loss is then calculated. Soaking of the materials in solutions with a pH equal to the pH of the human body is the initial simulation of the implant material being placed into the human body.

**Figure 4:**
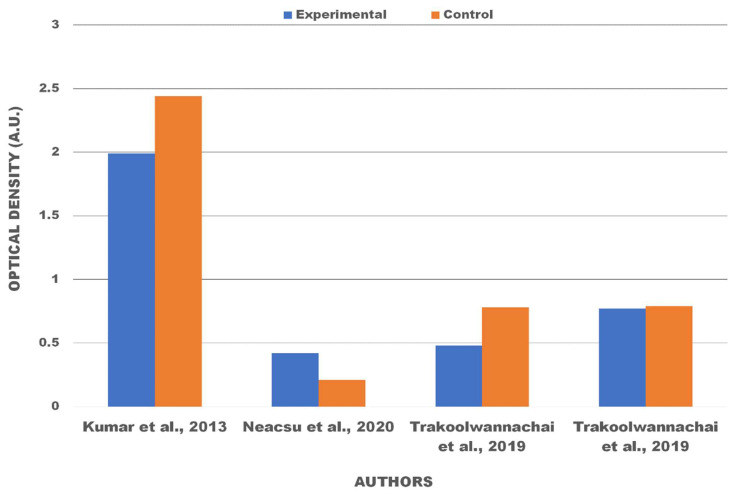
Optical density of materials after being cultured in various stem cells.

The cytotoxicity test, one of the biological evaluation and screening tests, uses tissue cells in vitro to observe the cell growth, reproduction, and morphological effects of medical devices [25]. The in vitro cytotoxicity test uses ISO 10993-5:2009 [26]. In this work, the limitation of cytotoxicity tests was based on the direct contact test. These methods specify the incubation of cultured cells in contact with a device and/or extracts of a device directly. Cytotoxic effects can be determined by either qualitative or quantitative means. Qualitative means are appropriate for screening purposes by examining the cells microscopically using cytochemical staining and general morphology. For quantitative means, the methods used and endpoints measured in cytotoxicity determination are based on cell growth, where the number of cells are quantified by objective means. Based on ISO 10993-5:2009, reduction of cell viability by more than 30% is considered a cytotoxic effect. Table 2 shows the results obtained from studies reporting on various kinds of stem cells used for cultures and the sample compositions of hydroxyapatite. There are two quantitative results obtained from the cytotoxicity tests—optical density and cell viability. Viability cells are the number of living cells in a population [65]. Viability assays were carried out with cultured cells from humans or animals. A viability assay is performed by observing the optical density of the absorbance value of the materials. Optical density testing is carried out to determine the number of growing cell colonies. Cell viability is the ratio of the optical density of the treatment and control materials in percentage units. Viability cells are shown as a percentage because it compares the value of optical density treatment and optical density control from eggshell-based hydroxyapatite. The statistical significance of the difference between the control and treatment groups was evaluated using a one-way analysis of variance (ANOVA) [45]. The quantification of viable cells was evaluated with the help of fluorescence microscopy [2,25,26], field emission scanning electron microscopy (FESEM) [52], and scanning electron microscopy [55]. The optical density test results from articles [2,20,49,50] are displayed in Figure 4 with a comparison of the control samples and treatment material variations, which can be used to determine the value of cell viability. The test results for cell viability from the studies are shown in Table 3 and Table 4. Table 3 shows viable cells using human stem cells while Table 4 shows viable cells using stem cells from various animals. Quantitative data are obtained from bar charts with standard deviation shown in the included articles.

## 4. Discussion

As shown in Table 2, several studies have produced eggshell-based hydroxyapatite materials that were mixed with other elements. Biocompatibility and bioactivity can be affected by the presence of minor elements that are present within the environment; for example, Mg^2+^, Al^3+^, Sr^2+^, Zn^2+^, K^+^, and Na can facilitate rapid bone regeneration [52], and minor impurities do not have any adverse effect on cell attachment and proliferation [32]. Ions play an important role in bone biology, cell spreading, and adhesiveness. Among the different ions, other than zinc and calcium, magnesium can contribute to cell spreading and adhesion. The significant amount of magnesium obtained from eggshell-derived materials explains the cell adhesion pattern. In addition, strontium is reported to have a beneficial effect on bone structural strength and osteoblast differentiation [51].

**Figure 5:**
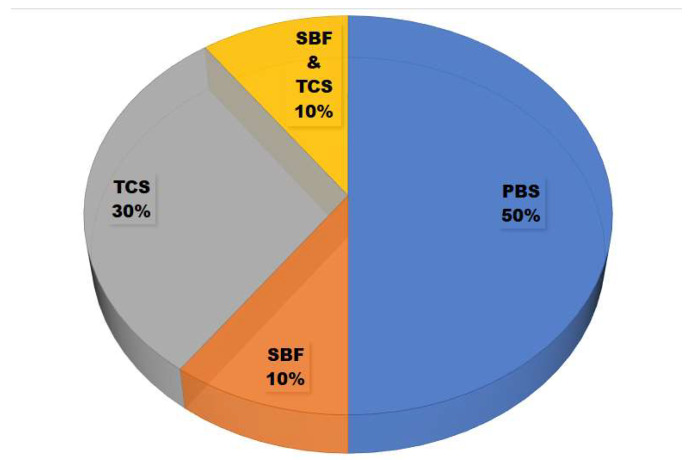
Solutions used for in vitro degradation tests to determine the weight loss of eggshell-based hydroxyapatite.

The increasing soaking times in the in vitro degradation testing are directly linear to the degradation rate and weight loss of the materials [43]. As shown in Figure 5, the solutions used for in vitro degradation testing in the selected studies were phosphate-buffered saline (PBS) [27,33,35,43,49], Tris-buffered saline (TBS) [28,29,30,31], and simulated body fluid (SBF) [28,29]. The third solution has the same function with ion concentrations nearly equal to human blood plasma, according to the conditions in the human body. TBS has an effective pH range of about 7.5–9.0, PBS has a pH range of about 6.0–8.0, and SBF has an ion concentration similar to human blood plasma. TBS does not contain ions, thus signifying the maximum solubility and minimum reprecipitation activity of the material [28].

**Figure 6:**
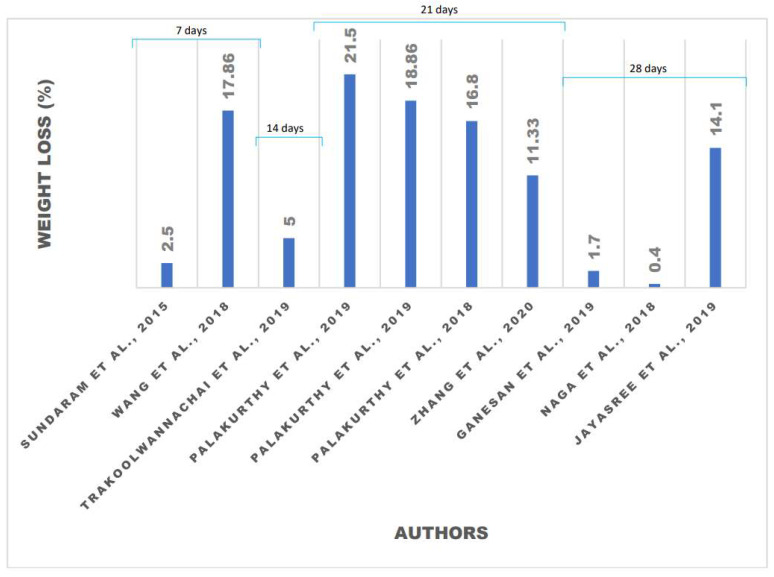
Weight loss of the eggshell-based hydroxyapatite after soaking for various times.

The degradation study was evaluated by measuring the calcium ion concentration, along with pH variation, of the medium and weight loss of the samples [27]. As shown in Figure 6, composite hydroxyapatite/zirconia toughed alumina (ZTA) has the smallest weight loss because ZrO_2_ and Al_2_O, which are the constituent elements of ZTA, are insoluble in water, thus limiting the diffusion of the solution in the composite material [56]. With the addition of other elements, such as composite HA/polycaprolactone (PCL), which is a type of polymer [49], the percentage of weight loss is also not very significant. The degradation process only occurs on the surface of the material [49]. This is because PCL absorbs water on the surface, so that after soaking for a few days, the materials gain weight [35]. When involving polymers in the manufacturing of composites, several factors must be considered, such as polymer hydration, breaking of monomer-bonds, polymer degradation, and diffusion of the solutions used. Consideration of the hydroxyapatite composition is needed to facilitate the filtration of water into the composite matrix [49]. Ganesan et al. (2019) conducted research on eggshell-based hydroxyapatite and the weight loss that occurred [27]. The process of in vitro degradation of the hydroxyapatite material for 28 days resulted in a weight loss that was less than 3%. This occurred because the sintering temperature was too high; thus, the hydroxyapatite changed into a secondary phase, namely β-TCP. This secondary phase caused the rate of the material weight loss to slow [27].

The addition of other elements to hydroxyapatite as a composite does not always reduce the weight loss of the material. The weight loss that occurred in the eggshell-based hydroxyapatite indicates these would be adsorbed in the human body. When adding selenite to hydroxyapatite, the material loses a significant amount of weight because selenite ions are released during the in vitro degradation. Increasing the amount of selenite in the composition also increases weight loss [43]. The addition of rice husk ash (RHA), which produces the element wollastonite, causes high weight loss. Weight loss that is too high causes the pH to be high as well, thus limiting clinical applications [28]. It is necessary to add other elements that are safe for medical applications to stabilize the weight loss of hydroxyapatite–wollastonite composites, including zirconia (Zr) [31] and argentum (Ag) [30], as well as the selection of the solutions used [35]. With the same soaking time and solution, zirconia is able to reduce the weight loss of materials. Furthermore, the use of the SBF solution can also reduce the weight loss of the material because hydroxyapatite settles on the surface of the SBF solution [28].

The degradation rate is related to the bioactivity of the material [29]. The degradation process is affected by several factors, such as powder crystallinity, grain size, microstructure, and the surface area of the material [31]. Connectivity between the grains also affects the process of material degradation [29]. The main phase of hydroxyapatite shows a high degradation rate [27]. Eggshell-based hydroxyapatite has a higher weight loss with a smaller crystal size. The ions produced from the eggshell also affect the weight loss of the materials [33]. It is not possible to stimulate cellular proliferation if low ionic concentrations result from very slow dissolution rates [28]. The pH of the medium increases for up to 7 days due to the release of OH ions from the samples by dissolution in the soaking medium. Subsequently, the pH value decreases, which may be assumed to be due to the deposition of apatite that consumes OH ions. Sundaram et al. conducted an experiment that showed eggshell-based hydroxyapatite prepared with polycaprolactone (PCL) nanocomposite film had a larger weight loss than other materials. This organic–inorganic composite was found to be suitable for bone tissue engineering [35].

In vitro cell culture testing provides information on the biocompatibility of the material [11]. There are two types of stem cells used for cultured in cytotoxicity test by optical density results, i.e. stem cells from humans [2,49,50], shown in Table 3, and stem cells from animals [11], shown in Table 4. Using cell culture methods can also stimulate cell proliferation [2]. The stem cells are usually used as permanent cells because they are similar to human bone osteoblasts [49]. If the optical density increases, the surface area also increases for cell adhesions. Cell adhesions provide advantages for growth and cell viability [50]. Materials that use eggshell-based hydroxyapatite have good optical density results because the absorbance for all porous scaffolds higher than control due to the high surface area determined by the porous morphology and the bioactive properties of calcium phosphate [2]. As shown in Figure 4, it was found that the material culture in stem cells does not cause cytotoxicity.

Materials for bone tissue engineering applications must be used without provoking any immune response in the human body [45]. Cell toxicity is one of the challenges related to the application of bone tissue engineering and the manufacturing of new biomaterials [14]. Cytotoxicity testing is used to determine metabolic function and cell health in the bone tissue environment [26]. One of the factors measured with cytotoxicity testing is cell viability. Viable cells are used to examine cell responses and determine the interactions between the cells in biomaterials [54]. Viable cells were shown as a percentage comparison for the value of optical density treatment and optical density control from eggshell-based hydroxyapatite. The interactions between cells and samples have an effect on growth and differentiate cells that affect bone growth [65]. Furthermore, cell attachment is an early indicator of the interaction between cell proliferation and cell differentiation [54].

$$\mathrm{Viability}\;\mathrm{Cells}=\frac{\left[\mathrm{OD}\;\mathrm{test}\right]}{\left[\mathrm{OD}\;\mathrm{control}\right]}\times 100\%$$

ISO 10993-5:2009 states that for in vitro cytotoxicity testing, if the percentage of viable cells is greater than 70% in the control group for the minimum concentration, the material is considered noncytotoxic [31]. It means that there should not be a decrease of viable cells of more than 30% [26]. In the analysis of the studies we conducted, the cultured cells came from humans and animals, including mice [27,35,40], rats [25,33,36], and monkeys [41,42]. As shown in Table 3 and Table 4, with the varieties of cell cultures used, all studies obtained viable cell results of more than 70% with a variety of materials, either pure eggshell-based hydroxyapatite or composites of eggshell-based hydroxyapatite and other materials.

The addition of Si and Mg to the hydroxyapatite composite can increase the viability and proliferation of cells in osteoblasts [55]. Yilmaz et al. [47] also tested HA/CS/GO composites, in which the addition of graphite was shown to increase the osteoconductivity of the materials. In addition, zirconia [31] and wollastonite compounds obtained from rice husk ash also did not have a biological cell toxicity effect [30]. In eggshell-based hydroxyapatite cement, viable cells reached about 110%, and the cell culture adhered to and spread on the sample surface because eggshell-based cement had smaller and densely packed crystals causing initial high compressive strength [33]. Egg white protein, which acts as a hydrogel, has benefits for osteogenic differentiation cells and increases bone regeneration when mixed with eggshells as hydroxyapatite [64]. Eggshell results in cell viability decreasing by less than 5% because CaO as a constituent of eggshell-based hydroxyapatite has high cytotoxicity [41].

Viable cells are affected by a few indicators, such as the sintering temperature [41], composition and size of the particles [45], morphology, porosity, and surface energy [35]. The sintering temperature affects the particle size and crystallinity of the material, where a higher sintering temperature is proportional to the crystallinity of the particles [41]. Viable cells decrease when heating the material to a higher temperature [25]. Cell activity and improved calcium release also occur in materials with low crystallinity [54]. As shown in Table 4, among the different calcium carbonate sources, eggshell-derived hydroxyapatite promotes the best cell adhesion and proliferation, which are comparable with those of pure hydroxyapatite but without the formation of clusters [51]. Compared with fish bones, fish scales, cuttlefish bone, and synthetic calcium carbonate, eggshell-based hydroxyapatite showed improved cell survival rate, because eggshell contains calcium oxide (CaO), which has high cytotoxicity [41]. Cuttlefish bone and synthetic calcium carbonate with round grains have poor cell adhesions [51]. However, in general, hydroxyapatite, which is made from various materials, showed biocompatibility and their application as implants for bone regeneration [36]. The use of eggshells as a raw material can sufficiently reduce processing costs [26].

In this work, several tests regarding the biocompatibility of eggshell-based hydroxyapatite have been summarized in the last ten years. The result of in vitro degradation and cytotoxicity tests are displayed by meta-analysis in the tables and figures; these contain the weight loss of eggshell-based hydroxyapatite, optical density, and the viability of cells using stem cells from human and animals. There is still need for further clinical research to ensure the biocompatibility of eggshell-based hydroxyapatite and its use in bone tissue engineering.

## 5. Conclusions

This literature review summarized the reported biocompatibility of eggshell-based hydroxyapatite by focusing on two aspects: in vitro degradation and cytotoxicity tests from various studies. Eggshell-based hydroxyapatite is an alternative biomaterial that can be useful for reducing natural waste. Biological testing is needed to determine the biocompatibility of implant materials. The results of in vitro degradation and cytotoxicity tests have proven that materials that use eggshells to formulate hydroxyapatite have significant biocompatibility. There can be a decrease in the weight loss of the material when soaking in solutions with the same pH stability as the human body, together with an appropriate cell response when the material is cultured with stem cells that have a similar structure to human bone. Continued research to ensure the biocompatibility of eggshell-based hydroxyapatite and its use as a candidate for bone tissue engineering can be a further opportunity for meta-analysis.

## Figures and Tables

**Table 1:** In vitro degradation test data based on various soaking solutions and times.

No	Authors	Year	Sample Composition	Soaking	
				Solutions	Days
1	Zhang et al. [31]	2020	Eggshell-based hydroxyapatite/ rice husk ash (RHA) + zirconia (Zr)	TBS	21
2	Jayasree et al. [33]	2019	Eggshell-based hydroxyapatite cement/ brushite cement	PBS	28
3	Palakurthy et al. [29]	2019	Eggshell-based hydroxyapatite	TBS	21
4	Trakoolwannachai et al. [49]	2019	Eggshell-based hydroxyapatite/ polycaprolactone (PCL)	PBS	14
5	Ganesan et al. [27]	2019	Eggshell-based hydroxyapatite	PBS	28
6	Palakurthy et al. [30]	2019	Eggshell-based hydroxyapatite/ rice husk ash (RHA)	TBS	21
7	Palakurthy et al. [28]	2018	Eggshell-based hydroxyapatite/ rice husk ash (RHA)	TBS and SBF	21
8	Wang et al. [43]	2018	Eggshell-based hydroxyapatite/ selenium (Se)	PBS	7
9	Naga et al. [56]	2018	Eggshell-based hydroxyapatite/ zirconia-toughened alumina (ZTA)	SBF	28
10	Sundaram et al. [35]	2015	Eggshell-based hydroxyapatite/ polycaprolactone (PCL)	PBS	7

**Table 2:** In vitro cytotoxicity test data based on various stem cells used for the culture.

No	Author	Year	Sample Composition	Various Stem Cells
1	Jahangir et al. [41]	2021	Eggshell-based hydroxyapatite; fish bone-based hydroxyapatite; fish scale-based hydroxyapatite	African Green Monkey Kidney Epithelial Cells
2	Tram et al. [54]	2021	Eggshell-based hydroxyapatite	Osteoblastic—Cell Line MC3T3-E1
3	Cestari et al. [51]	2021	Eggshell-based hydroxyapatite; cuttlefish bone-based hydroxyapatite; mussel shell-based hydroxyapatite	Human Lung Fibroblast Cell Line (MRC5)
4	Sultana et al. [42]	2021	Eggshell-based hydroxyapatite	African Green Monkey Kidney Cell
5	Zhang et al. [31]	2020	Eggshell-based hydroxyapatite/ rice husk ash (RHA)	Human Osteosarcoma MG-63 Cells
6	Horta et al. [26]	2020	Eggshell-based hydroxyapatite	Dental Pulp Stem Cells (DPSCs)
7	Holloman et al. [52]	2020	Eggshell-based hydroxyapatite; littleneck clam shell-based hydroxyapatite; quahog clam shell-based hydroxyapatite	Human Osteoblast Cells
8	Muthu et al. [40]	2020	Eggshell-based hydroxyapatite	L929 Cell Line (mouse fibroblast)
9	Neacsu et al. [2]	2020	Membrane Eggshell-based hydroxyapatite/bovine/chitosan/gel	GM0047 Amniotic Fluid Stem Cell Line
10	Oral et al. [48]	2020	Eggshell-based hydroxyapatite	Human Bone Cell (hFOB)
11	Ingole et al. [37]	2019	Eggshell-based hydroxyapatite	Human Bone-Derived Osteoblasts (hFOB)
12	Prieto et al. [55]	2019	Eggshell-based hydroxyapatite/ silicon (Si)/PLGA	Human Osteoblast Cell Systems
13	Li et al. [25]	2019	Eggshell-based hydroxyapatite	Rat Bone-Marrow-Derived Mesenchymal Stem Cells
14	Huang et al. [64]	2019	Eggshell-based hydroxyapatite and egg white	Human Dental Pulp Stem Cells (hDPSCs)
15	Patel et al. [45]	2019	Eggshell-based hydroxyapatite	Human Osteocyte Cells
16	Trakoolwannachai et al. [50]	2019	Eggshell-based hydroxyapatite/chitosan	Human Osteosarcoma Cells (Saos-2)
17	Jayasree et al. [33]	2019	Eggshell-based hydroxyapatite cement/brushite cement	L6 and MG63 Cells
18	Trakoolwannachai et al. [49]	2019	Eggshell-based Hydroxyapatite/Polycaprolactone (PCL)	Human Osteosarcoma Cells (Saos-2)
19	Ganesan et al. [27]	2019	Eggshell-based Hydroxyapatite	L929 Cells (mouse fibroblast)
20	Patel et al. [44]	2019	Eggshell-based Hydroxyapatite	Bone Marrow-Derived Mesenchymal Stem Cells (BMSCs)
21	Roopavath et al. [32]	2019	Eggshell-based Hydroxyapatite	Human Adipose-Derived Mesenchymal Stem Cells (ADSCs)
22	Palakurthy et al. [28]	2018	Eggshell-based Hydroxyapatite/ Rice Husk Ash (RHA)	Human Osteosarcoma MG-63 Cells
23	Nga et al. [53]	2018	Eggshell-based Hydroxyapatite	MEM with SBF
24	Wang et al. [43]	2018	Eggshell-based Hydroxyapatite/ Selenium (Se)	Whole Blood of Forty Healthy Individuals
25	Yılmaz et al. [47]	2018	Eggshell-based Hydroxyapatite/Graphite/Chitosan	MC3T3-E1 Cells
26	Ingole et al. [36]	2017	Eggshell-based Hydroxyapatite; commercial HA	Ham’s F12
27	Arslan et al. [46]	2017	Eggshell-based Hydroxyapatite/Hair Keratin/Jellyfish Collagen	Human Amniotic Mesenchymal Stem Cells (AMSCs)
28	Choudhary et al. [38]	2016	Eggshell-based Hydroxyapatite	Human Bone Marrow Stromal Cells (hMSCs)
29	Ingole et al. [14]	2016	Eggshell-based Hydroxyapatite	Human Bone-Derived Osteoblasts (hFOB)
30	Sundaram et al. [35]	2015	Eggshell-based Hydroxyapatite/Polycaprolactone (PCL)	Fibroblast Cell Line NIH-3T3 and Osteoblast Cell Line MG-63
31	Kattimani et al. [39]	2014	Eggshell-based Hydroxyapatite	Human Osteoblast Cells
32	Kumar et al. [20]	2013	Eggshell-based Hydroxyapatite	3T3-L1 cells (mouse fibroblast)
33	Mondal et al. [34]	2012	Eggshell-based Hydroxyapatite; Fish Bone- based Hydroxyapatite; Bovine Bone-based Hydroxyapatite	RAW Cell Lines

**Table 3:** Results for viable cell of cytotoxicity testing using human stem cells.

No.	Author	Year	Materials		Composition	Results	
			Eggshell	Composite		Day	Viability Cells (%)
1	Palakurthy et. al. [29]	2020	-	ν	Eggshell based Hydroxyapatite/Rice Husk Ash (RHA)	2	>70
2	Horta et. al. [26]	2020	ν	-	Eggshell based Hydroxyapatite	1	98.9
3	Ingole et. al. [37]	2019	ν	-	Eggshell based Hydroxyapatite	1	>95
4	Prieto et. al. [55]	2019	-	ν	Eggshell based Hydroxyapatite/Silicon (Si)/PLGA	8	>80
5	Huang et. al. [64]	2019	ν	-	Eggshell based Hydroxyapatite and Eggwhite	2	>70
6	Patel et. al. [45]	2019	ν	-	Eggshell based Hydroxyapatite	1	>90
7	Palakurthy et. al. [29]	2018	-	ν	Eggshell based Hydroxyapatite/Rice Husk Ash (RHA)	2	>70
8	Arslan et. al. [46]	2017	-	ν	Eggshell based Hydroxyapatite/Hair Keratin/Jellyfish Collagen	21	>90
9	Ingole et. al. [14]	2016	ν	-	Eggshell based Hydroxyapatite	1	>100
10	Sundaram et. al. [35]	2015	-	ν	Eggshell based Hydroxyapatite/Polycaprolactone (PCL)	7	>96

**Table 4:** Results for viable cell of cytotoxicity testing using stem cells from animals.

No.	Author	Year	Materials		Composition	Results	
			Eggshell	Composite		Day	Viability Cells (%)
1	Jahangir et. al. [41]	2021	ν	-	Eggshell based Hydroxyapatite; Fish Bone based Hydroxyapatite; Fish Scales based Hydroxyapatite	2	>95
2	Tram et. al. [54]	2021	ν	-	Eggshell based Hydroxyapatite	7	>70
3	Sultana et. al. [42]	2021	ν	-	Eggshell based Hydroxyapatite	1	>80
4	Muthu et. al. [40]	2020	ν	-	Eggshell based Hydroxyapatite	1	>96
5	Jayasree et. al. [33]	2019	-	ν	Eggshell based Hydroxyapatite cement/Brushite cement	3	>100
6	Li et. al. [25]	2019	ν	-	Eggshell based Hydroxyapatite	7	>80
7	Ganesan et. al. [27]	2019	ν	-	Eggshell based Hydroxyapatite	21	80
8	Yılmaz et. al. [47]	2018	-	ν	Eggshell based Hydroxyapatite/Graphite/Chitosan	1	>70
9	Ingole et. al. [36]	2017	ν	-	Eggshell based Hydroxyapatite; HA comercially	7	>95

## Data Availability

The data presented in this study are available from the corresponding author.
